# A Dual-Stage Multi-Reference Spectral-Subtraction Compensation Denoising Algorithm for Measuring Explosion Electromagnetic Radiation Field Strength

**DOI:** 10.3390/s26196054

**Published:** 2026-09-24

**Authors:** Yu Hao, Xuchao Pan, Lin Zhou, Wei Du, Siwei Wang, Haoran Sun, Xin Su, Mengli Xie, Junjie Jiao, Zhong Fang, Yong He

**Affiliations:** School of Mechanical Engineering, Nanjing University of Science and Technology, Nanjing 210094, China; haoyu@njust.edu.cn (Y.H.);

**Keywords:** explosion electromagnetic radiation, signal processing, spectral subtraction, electric field-strength measurement

## Abstract

Explosion electromagnetic radiation (EEMR) is important for studying explosive detonation, but environmental interference and system frequency-response characteristics influence its electric-field-strength characteristics. This study analyzes EEMR and proposes a dual-stage multi-reference spectral-subtraction compensation denoising (DMSCD) algorithm for field-strength measurement. DMSCD compares signal and pre-sampling noise spectra to identify and attenuate noise-dominant frequency components, and then applies Wiener-gain suppression to overlapping residual noise. To evaluate DMSCD, six baseline algorithms are selected. For 90 EEMR segments from 10 g Φ 20 mm × 20 mm RDX charges, DMSCD reduced the average peak-to-peak error to 74%, lower than that without denoising. Its reconstructed spectra retained clear signal peaks near 40, 126, 221, 270, 388, and 675 MHz, with 160–337 MHz as the main frequency component. Compared with the baseline algorithms, DMSCD more effectively suppressed noise in the frequency domain while preserving time-domain pulse characteristics. In the processing of EEMR, DMSCD can effectively utilize the signal characteristics of the pre-sampling segment to identify and suppress the influence of broadband noise, achieving good denoising performance in the time–frequency domain.

## 1. Introduction

Explosion electromagnetic radiation (EEMR) is a complex phenomenon involving the outward radiation of electromagnetic fields during an explosion [1,2,3]. Its sources include explosively generated plasma [4,5,6,7,8,9,10,11,12,13], asymmetric scattering of detonation products [14,15,16,17,18,19], the formation of air shock waves [20,21,22,23,24,25], and charge separation in explosion plasma [26,27,28]. Its time–frequency characteristics are closely related to the evolution of the explosion process, and it has become a research focus in the field of detonation [2,13]. The EEMR spectrum may be distributed over a broad range from tens of kilohertz [7] to hundreds of megahertz [2,13,29]. At the same time, because explosion tests require open outdoor protective sites, signal measurements are usually disturbed by environmental electromagnetic signals such as radio waves [30,31]. Broadband transient electromagnetic measurement systems are also sensitive to bandwidth limitations, nonideal frequency responses, and noise during signal reconstruction [32,33]. This imposes complex requirements on signal processing: the energy in noise frequency bands must be separated and eliminated, and the noise energy in overlapping frequency bands must be suppressed.

In the past, wavelet denoising (WD) has mainly been used to process EEMR [2,31,34,35]. WD uses a preset wavelet basis function to decompose the original waveform into a multilayer structure and suppresses the energy in noise frequency bands by applying threshold processing to the decomposition results [36]. Its denoising performance is affected by the selection of the wavelet basis function, while the selection of thresholds for the decomposition results at each wavelet level mainly relies on experience, making it difficult to adapt to different signal characteristics [35,36]. Therefore, in actual signal analysis, only comparative time–frequency analysis under the same test environment can usually be performed [24,37], and there is a lack of capability to obtain the true electric field-strength signal.

To obtain more accurate EEMR electric field-strength waveforms and spectral distributions, this paper analyzes the signal characteristics and test conditions, proposes a dual-stage multi-reference spectral-subtraction compensation denoising (DMSCD) algorithm, and selects six baseline algorithms for comparative performance evaluation. DMSCD uses the stationarity of the background noise and compares the signal and noise spectra to identify and progressively suppress noise amplitudes in the frequency domain, thereby suppressing noise while retaining signal characteristics. DMSCD uses the pre-sampling stage for noise estimation, distinguishes noise components, and independently denoises individual frequency bins, providing high frequency resolution and denoising accuracy.

## 2. Materials and Methods

### 2.1. Characteristic Analysis of EEMR Signals

To analyze the characteristics of EEMR signals, EEMR measurement experiments were first conducted. The measurement-system layout is shown in Figure 1.

The experimental system used an A.H. Systems SAS-565H loop antenna (A.H. Systems, Inc., Chatsworth, CA, USA), a Schwarzbeck EFS9218 biconical antenna (Schwarzbeck Mess-Elektronik OHG, Schönau, Germany), and a Schwarzbeck FSHPE single-axis E-field antenna (Schwarzbeck Mess-Elektronik OHG, Schönau, Germany) as receiving antennas. A Tektronix 64B oscilloscope (Tektronix, Inc., Beaverton, OR, USA) was used to acquire the signals, with a sampling rate of 50 GS/s and a bandwidth of 2.5 GHz. Triggering the oscilloscope simultaneously triggered the Phantom TMX 6410 high-speed camera (Vision Research, Inc., Wayne, NJ, USA). The explosives were RDX charges with dimensions of Φ 20 mm × 20 mm, a density of 1.6 g/cm^3^, and a mass of 10 g. The charges were suspended vertically at a height of 1.5 m above the ground, and all antennas were placed 5 m from the explosion center. The choice of explosive does not affect the algorithm evaluation. The same RDX geometry was used in every experiment to ensure consistency and comparability and to reduce additional system errors caused by different experimental settings. Six experiments were conducted. Each experiment contained a different number of EEMR signals, and 90 EEMR signal segments were obtained. A typical EEMR signal is shown in Figure 2. Figure 2a shows the measured time-domain waveform, Figure 2b shows the FFT decomposition result of Figure 2a, and Figure 2c shows the spectral characteristics during the pre-sampling stage of the same waveform, which can be regarded as the actual noise spectrum of the measurement system.

As can be seen from Figure 2a, the example signal is a wave packet with an obvious steep rise and slow decay and has superimposed double-exponential and oscillatory waveform characteristics. In the frequency domain, the signal and noise have different spectral distribution characteristics. Moreover, because the antennas have different response curves, the spectra obtained with them have approximately the same spectral peaks, but the relative relationships between the amplitudes differ.

To further analyze the frequency characteristics of the signal and noise and reduce the random test errors and equipment-induced statistical errors in the frequency-domain characteristics, the FFT results of the signal and noise were statistically analyzed. Interpolation was used to obtain the amplitude-distribution characteristics of all frequency components from 0 to 3 GHz. To prevent differences in antenna gain from causing unequal weights, the spectrum of each segment was normalized by its maximum amplitude, and box-and-whisker plots were then drawn at each frequency point. Finally, the mean, median, upper and lower quartiles, and upper and lower whiskers were plotted as spectral-characteristic statistical plots, thereby obtaining the main spectral distribution characteristics of the signal and noise, as shown in Figure 3.

Figure 3 shows that the signal and noise segments have different spectral distribution characteristics. Figure 3a shows that the signal has spectral peaks at 0, 48, 125, 215, 271, 390, 664, 756, 872, 937, 1172, and 1563 MHz. The energy gradually decreases above 1 GHz, while the amplitudes above 2.5 GHz rapidly return to zero because of the system bandwidth. Figure 3b shows that the noise has spectral peaks at 0, 196, 391, 586, 778, 879, 941, 1172, 1367, and 1563 MHz. A comparison of Figure 3a,b shows that the noise spectral peaks have narrow bandwidths and dispersed frequency distributions. In the signal spectrum, corresponding amplitude increases can also be observed at the frequencies of the noise spectral peaks, and there are also many frequency components at which the noise amplitude is low while the signal amplitude is high. Therefore, for the denoising of EEMR signals, the noise frequency bands need to be correctly distinguished and removed, while the noise energy in the overlapping frequency bands also needs to be suppressed to retain the frequency characteristics of the signal.

The noise characteristics can be obtained from the pre-sampling stage of each measurement. Time–frequency analysis of a single measurement signal directly shows the stability of the noise, as illustrated in Figure 4a.

Figure 4a shows that, apart from the intervals in which signals clearly occur, the noise spectral distribution is relatively stable. Integrating the spectrum of each time window over frequency bands and taking the ratio to the mean power during the pre-sampling stage give Figure 4b. The fluctuation throughout the pre-sampling stage is below 1.5 dB, whereas the fluctuation at the signal time exceeds 8 dB. The mean difference between the maximum and minimum values over all pre-sampling intervals is 1.81 dB. Therefore, the pre-sampling noise characteristics can represent the noise characteristics of the entire measurement process.

### 2.2. DMSCD Algorithm

Based on the differences between the signal and noise spectra identified above, this paper proposes DMSCD for EEMR field-strength measurement. The first stage attenuates noise-dominant frequency bins and performs constrained spectral compensation, whereas the second stage applies a noise-reference-based Wiener-type spectral gain to suppress residual noise in overlapping frequency bands.

Let the one-dimensional discrete voltage signal acquired by the oscilloscope at a sampling rate $f_{s}$ be x[n]=s[n]+v[n], where s[n] is the original voltage waveform of the signal segment, v[n] is the background noise and external interference, and n=0,1,…,N−1. DMSCD first extracts the time window containing the pulse to be processed and calculates its complex spectrum, $X_{0}\left[k\right]=\mathrm{FFT}\left\{x\left[n\right]\right\}$, with the corresponding discrete frequency $f_{k}=k/\left(N\Delta t\right)$, where $\Delta t=1/f_{s}$. The pre-sampling interval $\Omega_{N}$ before the measurement-system trigger can be approximately regarded as a noise interval. A noise reference segment with the same length as the signal to be processed is randomly extracted from this interval. The first-stage reference segments are denoted by $v_{m}\left[n\right]$, and their spectra are $V_{m}\left[k\right]=\mathrm{FFT}\left\{v_{m}\left[n\right]\right\}$, where $m=1,\ldots ,M_{1}$.

The first stage performs noise-dominance classification, attenuation, and constrained spectral compensation at each frequency bin. Let $X^{\left(0\right)}\left[k\right]=X_{0}\left[k\right]$. For the *m*-th noise reference segment, the following update is used:

(1) $$\begin{array}{cc} D_{m}\left[k\right] & =I\{|V_{m}\left[k\right]|>T_{s}|X^{\left(m-1\right)}\left[k\right]|\},\\ {\tilde{X}}^{\left(m\right)}\left[k\right] & =X^{\left(m-1\right)}\left[k\right]R_{s}^{-D_{m}\left[k\right]},\\ \rho_{m}\left[k\right] & =\frac{|X_{0}\left[k\right]|}{|{\tilde{X}}^{\left(m\right)}\left[k\right]|+\varepsilon },\\ \Gamma_{m}\left[k\right] & =\min\{R_{f},\max\left(1,\rho_{m}\left[k\right]\right)\},\\ X^{\left(m\right)}\left[k\right] & ={\tilde{X}}^{\left(m\right)}\left[k\right]\Gamma_{m}\left[k\right]. \end{array}$$

Here, I{·} is the indicator function, $T_{s}$ is the noise-dominance classification threshold, $R_{s}>1$ is the attenuation factor, $R_{f}\geq 1$ is the compensation factor, and ε is a very small positive number used to avoid division by zero. If $D_{m}\left[k\right]=1$, the spectral amplitude at the *k*-th frequency bin is reduced by a factor of $R_{s}$; otherwise, it remains unchanged. After the sequential updates using the $M_{1}$ noise reference segments, the first-stage output is $X_{I}\left[k\right]=X^{\left(M_{1}\right)}\left[k\right]$. Neither the attenuation nor the constrained compensation increases the amplitude beyond its original value, so $0\;\leq \;|X^{\left(m\right)}\left[k\right]|\leq \;|X_{0}\left[k\right]|$. This constraint reduces the risk of amplifying errors and avoids excessive weakening of the signal-containing frequency bins.

For the second stage, another set of $M_{2}$ noise reference segments is extracted from the pre-sampling interval, and their spectra are denoted by $V_{\ell }\left[k\right]$. The noise power spectrum is estimated as $P_{N}\left[k\right]={\mathrm{median}}_{\ell =1,\ldots ,M_{2}}{\left|V_{\ell }\left[k\right]\right|}^{2}$, from which the following spectral gains are obtained:

(2) $$\begin{array}{cc} G_{2}\left[k\right] & =\frac{|X_{I}{\left[k\right]|}^{2}}{|X_{I}{\left[k\right]|}^{2}+\alpha_{2}P_{N}\left[k\right]+\varepsilon },\\ G_{2c}\left[k\right] & =\min\{1,\max\left\{G_{2}\left[k\right],g_{\min,2}\right\}\},\\ {\bar{G}}_{2}\left[k\right] & =\min\left\{1,\max\left\{\frac{1}{L_{2}}\sum_{r=-h}^{h}G_{2c}\left[\left(k+r\right)\;\mathrm{mod}\;N\right],g_{\min,2}\right\}\right\},\\ X_{\mathrm{II}}\left[k\right] & ={\bar{G}}_{2}\left[k\right]X_{I}\left[k\right]. \end{array}$$

Here, $\alpha_{2}$ controls the contribution of the estimated noise power, $g_{\min,2}$ is the lower bound of the gain, and $L_{2}=2h+1$ is the length of the circular smoothing window. Applying the power-domain gain to the complex spectrum retains the phase of $X_{I}\left[k\right]$. This noise-reference-based Wiener-type spectral gain is related to spectral-subtraction estimators but does not directly subtract one spectrum from another [38,39,40,41,42].

The final DMSCD output spectrum is $\hat{X}\left[k\right]=X_{\mathrm{II}}\left[k\right]$, and the time-domain denoising result is $\hat{x}\left[n\right]=\mathrm{IFFT}\left\{\hat{X}\left[k\right]\right\}$. If the DC offset needs to be removed, $\hat{X}\left[0\right]=0$ is set. The complete algorithm flow is shown in Figure 5.

### 2.3. Implementation Details

To obtain the electric field-strength waveform, the field strength is calculated according to the Friis transmission formula:

(3) $$\tilde{E}\left(f\right)=\tilde{V}\left(f\right)10^{\left[\mathrm{AF}\left(f\right)+L_{\mathrm{dir}}\left(f\right)-S_{12,\mathrm{dB}}\left(f\right)-10\log_{10}\eta_{p}\left(f\right)\right]/20},\;f\in \Omega_{\mathrm{valid}},$$

where $\tilde{E}\left(f\right)$ is the complex spectrum of the electric field strength, $\tilde{V}\left(f\right)$ is the complex voltage spectrum, AF(f) is the calibrated antenna factor (dB/m), $L_{\mathrm{dir}}\left(f\right)$ is the directional attenuation, $S_{12,\mathrm{dB}}\left(f\right)$ is the cable S12 parameter (dB), $\eta_{p}\left(f\right)$ is the polarization matching efficiency, and $\Omega_{\mathrm{valid}}$ is the valid frequency range. The specific system frequency-response characteristics are shown in Figure 6.

It can be seen that the three antennas have different frequency-response characteristics within their valid frequency ranges.

## 3. Results and Discussion

All 90 EEMR segments were processed using one fixed parameter set. For DMSCD, the parameters were $M_{1}=194$, $T_{s}=1.03$, $R_{s}=3.51$, $R_{f}=1.22$, $M_{2}=188$, $\alpha_{2}=1.23$, $g_{\min,2}=0.0005$, $L_{2}=7$, and h=3.

Figure 2 shows that the amplitudes of the signals received by the three antennas differ. The explosive charge in the experiment was suspended vertically, and the charge was cylindrical, so both detonation and radiation had axisymmetric characteristics. Therefore, although the three antennas had different frequency responses, the electric field strengths of the same data segment should be consistent.

Figure 7 shows the processing results obtained by the different antennas in the same  experiment.

Figure 7a and Figure 7b show that the peak-to-peak values of the electric field-strength waveforms from the three antennas without denoising were 4.53, 2.33, and 1.81 V/m, respectively, and the maximum peak-to-peak error was 149%. The spectral distributions also showed large differences. The amplitude of the loop antenna was significantly greater than those of the other two antennas, while above 705 MHz, the spectral amplitudes of the loop antenna and the biconical antenna increased again. This was most likely because the high-frequency equipment noise floor was erroneously amplified by the large magnitude of the field-reconstruction frequency response.

As can be seen from Figure 7c, the reconstructed electric field-strength peak-to-peak values obtained from the three antennas using DMSCD denoising were 1.49, 1.87, and  1.68 V/m, respectively, and the maximum peak-to-peak error was 25.6%. The peak errors may originate from random errors such as complex reflections at the test site, cable coupling, antenna installation angles, and measurement-distance errors.

Figure 7d shows the spectral distributions of the three antennas. It can be seen that the spectra of the biconical antenna and the single-axis E-field antenna are highly consistent within the range of 0–364 MHz. Above 364 MHz, the amplitude of the single-axis E-field antenna is greater. The amplitudes measured by the loop antenna are poorly consistent with those measured by the other two antennas, but its spectral distribution range is basically consistent with those of the other two antennas. These spectral errors mainly originate from the different calibration ranges of the antennas themselves. The loop antenna has the smallest applicable frequency range, followed by the biconical antenna, while the single-axis E-field antenna has the largest applicable range. After the calibration range of an antenna is exceeded, its frequency response changes nonlinearly, causing large fluctuations in the calibration data themselves and consequently large errors.

Figure 7 shows that DMSCD can significantly improve the consistency of the reconstructed electric field-strength results, effectively suppress the energy in the noise frequency bands, and retain the frequency characteristics of the signal.

To further evaluate the performance of DMSCD, appropriate baseline algorithms need to be selected for comparison. Conventional time-domain smoothing and frequency-domain filtering cannot identify and suppress noise among multiple spectral peaks, whereas mode decomposition and feature extraction are more suitable for broadband signals with multiple spectral peaks, such as EEMR. Extracting the main frequency features through mode decomposition and then applying thresholds to suppress energy in the noise frequency bands can suppress noise while retaining signal features. Accordingly, variational mode decomposition (VMD) [43,44] and singular spectrum analysis (SSA) [45,46,47] were selected as baseline algorithms. Spectral subtraction (SS) [40,41] and Wiener filtering (WF) [38], whose principles are similar to those of DMSCD, were also included, along with WD [48], which is commonly used for EEMR processing, and the recently proposed adaptive wavelet denoising (AWD) method of Yang et al. [49]. The same waveforms were processed, and the results of the baseline algorithms are shown in Figure 8.

For VMD, the parameters used were K=34, α=12000, and τ=0.42, with a convergence tolerance of $4.96\times 10^{-6}$ and a maximum of 422 iterations. The center frequencies were randomly initialized, modes were selected using the kurtosis rule with a threshold of 5.78, the maximum computational length was 3750 points, and the DC component was removed. For SSA, the parameters used were a Hankel-window length of L=400, fixed-rank mode with r=49, a maximum computational length of 4096 points, and DC-component removal. SS used the mean power spectrum of 288 pre-sampling noise segments to estimate the noise power spectrum, an over-subtraction coefficient of 1.23, and a power-spectrum floor equal to 0.0005 times the input-signal power spectrum. WF used the mean power spectrum of 188 pre-sampling noise segments to estimate the noise power spectrum. Both algorithms removed the DC component. WD used the sym4 wavelet basis, hard thresholding, and a threshold scale factor of 1.3771455877. The decomposition level was set to 14, with the actual level limited by the input-signal length and the wavelet-filter length. The DC component was removed. AWD used the db9 wavelet basis, eight decomposition levels, and an amplitude scale of 1, without DC removal. Both wavelet algorithms used symmetric boundary extension.

As can be seen from Figure 8, the AWD method attenuates too much information, causing its time-domain waveform to become nearly invalid and excessively reducing the spectral energy. The other baseline methods generally show inaccurate frequency-domain identification and a high time-domain noise floor. DMSCD performs better in reducing the noise level and retaining the signal-frequency characteristics. Compared with its predecessor SS and WF methods, DMSCD also provides an improvement.

The peak-to-peak values of the reconstructed results obtained by each algorithm for the other 89 EEMR segments were further calculated. Valid data acquired simultaneously by different antennas in the same experiment were organized into one group, yielding 13 valid groups. The maximum and minimum peak-to-peak values within each group were selected to analyze the relative error, as shown in Table 1.

Because VMD suppresses the signals in some segments to nearly straight lines close to zero, these invalid results were excluded from the VMD statistics in the table. Table 1 shows that SS has the smallest average peak-to-peak error, followed by DMSCD. Although the peak-to-peak error of DMSCD is greater than that of SS, Figure 7 and Figure 8 show that DMSCD is more stable in the frequency domain and provides reconstructed field-strength results with better consistency.

Nevertheless, the reconstructed results still have large errors. Several factors contribute to the discrepancies in EEMR field-strength consistency. First, explosion experiments must be conducted at an impact-protection site with complex electromagnetic characteristics, and the measurement-system frequency response calibrated in the laboratory differs from that under actual measurement conditions. The antenna calibration range is 9 kHz–1 GHz; the spectral amplitudes at frequency bins outside this range were set to zero to prevent erroneous estimation, which also introduces errors. Second, the polarization characteristics of the explosion source are related to assembly and installation errors and to variations in explosive voids and density. Differences in polarization characteristics cause errors during field-strength reconstruction. Finally, because of limitations in site dimensions and signal amplitude, the antennas were placed 5 m from the explosion center. This distance does not satisfy far-field conditions for low-frequency signals, and the antennas are not co-located, also contributing to unstable measurement results. To obtain the most accurate results, the ideal EEMR test conditions are an open site without electromagnetic interference and measurement points located in the far-field region of the analyzed frequency band.

EEMR originates from explosively generated plasma. The plasma temperature generated by an explosion is approximately 1 eV, and the ionization degree of high-density explosion plasma is sensitive to temperature near this value. Because EEMR is proportional to the number of charged particles produced by ionization, the ionization-degree change corresponding to the maximum 164% discrepancy was converted using the Saha equation into an estimated temperature fluctuation of approximately 15.3%. EEMR is currently used mainly to study its correlations with detonation pressure and temperature; therefore, this discrepancy is within the acceptable range for that purpose.

Because the above errors are difficult to eliminate in actual implementation, the analysis of EEMR characteristics should be based on a comprehensive statistical analysis in the time and frequency domains. Based on the time-domain waveform analysis, this study further performs a statistical analysis of the spectra to obtain a more reliable evaluation. Figure 9 and Figure 10 show the spectral statistical results of DMSCD and all baseline algorithms.

Figure 9 is used to illustrate the denoising effect of DMSCD on the signal spectrum. Figure 9a shows the signal-spectrum statistics after DMSCD processing. A comparison of Figure 3 and Figure 9a shows that the spectral amplitudes at the frequency points near 586 and 1367 MHz, which exist only in the noise spectrum, are effectively eliminated. Near 48, 125, 271, and 664 MHz, where a signal distribution but no noise distribution exists, large spectral peaks are still retained. Among the spectral peaks shared by the signal and noise, the spectral amplitudes at the frequency points near 0, 756, 872, 937, 1172, and 1563 MHz are eliminated, whereas those near 215 and 390 MHz are retained. However, the spectral peaks are shifted relative to those of the original signal because the superposition of noise near an original true spectral peak causes the amplitude near the peak to exceed the true peak.

According to Equation (3), the signal denoised by DMSCD was reconstructed into electric field strength, and the frequency-domain statistical analysis is shown in Figure 9b. A comparison of Figure 9a,b shows that after electric field-strength reconstruction, the amplitudes of the spectral peaks change slightly and the differences among the spectral-peak amplitudes decrease, whereas the frequencies of the spectral peaks remain essentially unchanged, except that a new spectral peak appears at 560 MHz.

A comparison of Figure 9b,c shows that the spectral distribution without denoising tends to become uniform, making it difficult to distinguish the actual spectral-peak positions of the signal. This is because the energy in the noise frequency bands is amplified by the field-reconstruction frequency response. In the time-domain waveform, this situation results in a large background waveform amplitude. The larger the magnitude of the field-reconstruction frequency response, the more the low-amplitude noise is amplified. This characteristic can be seen in Figure 7a. In contrast, clear peaks can be observed in the electric field-strength spectrum after DMSCD denoising, and the spectral peaks in the median curve are more apparent than those in the other spectral curves. Moreover, because the noise is suppressed, the signal-to-noise ratio of the waveform increases significantly. This characteristic can also be seen in Figure 7c.

Figure 10 shows the denoising results of all baseline algorithms. A comparison of Figure 9a and Figure 10a,c shows that the trends of the SSA and DMSCD results are essentially consistent below 1 GHz. Above 1 GHz, although the SSA result also has an energy distribution, its amplitude is low, and the noise peak near 1563 MHz is not eliminated. VMD significantly suppresses low-amplitude modes and retains only the two largest main peaks, thereby losing detailed information. The spectrum in Figure 10c also shows that VMD does not effectively suppress the noise components.

A comparison of Figure 9b and Figure 10b,d shows that the shape of the SSA field-strength spectrum differs little from that without denoising and has no prominent spectral peaks, indicating a limited denoising effect. The main energy of the VMD field-strength spectrum is concentrated at 400–600 MHz, and amplitude information in other bands is lost, indicating over-suppression. This also leads to the previously mentioned cases in which the entire time-domain waveform is set to zero. Together with the waveforms in Figure 8a,c, these results show that the two baseline algorithms cannot effectively identify and suppress the noise components. In measurement systems with a large field-reconstruction frequency-response magnitude, the reconstructed field-strength waveforms contain large noise amplitudes. This is because the principles of the two baseline algorithms prevent them from using pre-sampling noise characteristics; they can only denoise according to the characteristics of the signal itself and therefore cannot effectively identify the noise frequency bands. Because the generation mechanism of EEMR is unclear and its signal characteristics are unknown, targeted parameter optimization cannot be performed, making most current algorithms unable to process EEMR signals effectively. By introducing pre-sampling signal characteristics, DMSCD can accurately suppress noise energy without relying on known signal characteristics. Figure 10e–j show that the frequency-domain results of SS, WF, and WD have some similarity to those of DMSCD. AWD, however, loses too much high-frequency information.

From the perspectives of peak values and spectral distributions, which are of particular interest in EEMR research, this section analyzes field-strength waveform consistency and spectral statistics. The results show that, when the signal characteristics are not yet clear, DMSCD balances noise suppression and signal preservation in EEMR processing and highlights signal characteristics in both the time and frequency domains. After field-strength reconstruction, the waveform peaks are relatively reliable and the spectral distribution characteristics are clear, meeting the algorithm’s design objectives. Compared with the baseline algorithms, DMSCD has strong denoising capability and applicability to EEMR.

To further demonstrate the denoising performance of DMSCD, the peak-to-peak relative error (PPRE), normalized root-mean-square error (NRMSE), and spectral relative error (SRE) of the denoised signals were quantitatively evaluated using constructed example waveforms. The ground-truth waveform was constructed using the following equation:

(4) $$s_{\mathrm{EEMR}}\left(t\right)=A\left(e^{-\alpha t}-e^{-\beta t}\right)\sum_{q=1}^{5}\sin\left(2\pi f_{q}t+\varphi_{q}\right),$$

where *A* is the amplitude-normalization coefficient, α and β control the decay and rise characteristics of the double-exponential envelope, and $f_{q}$ and $\varphi_{q}$ control the main frequency components and their phases. In the template used in this paper, $\alpha =5.0\times 10^{7}\;s^{-1}$ and $\beta =2.5\times 10^{8}\;s^{-1}$. The frequency components were 100 MHz, 110 MHz, 120 MHz, 130 MHz, and 1000 MHz, and the corresponding phases were $0^{\circ }$, $30^{\circ }$, $0^{\circ }$, $60^{\circ }$, and $90^{\circ }$, respectively. These parameters were selected randomly; however, to ensure a fair comparison among the algorithms, the constructed waveforms used the same parameters.

Uniform noise, normally distributed noise, and actual pre-sampling signals were added, and different signal-to-noise ratio levels and sampling rates were set to simulate various scenarios in actual measurements as far as possible. The constructed noisy signals were processed using DMSCD and several baseline algorithms, and the field strength was reconstructed using one set of actual antenna parameters. The error statistics relative to the true field strength are shown in Table 2. Statistical comparisons between DMSCD and each baseline used two-sided paired Wilcoxon signed-rank tests on the 200 pooled records (50 with uniform noise, 50 with Gaussian noise, and 100 with measured noise), with unadjusted p<0.05 indicating significance.

As shown in Table 2, DMSCD had a lower mean NRMSE and SRE than all six baseline algorithms, with significant paired differences (maximum Wilcoxon p=0.00604<0.05). DMSCD had a lower mean PPRE than VMD, SSA, WD, and AWD but slightly higher mean PPRE than SS and WF; these paired differences were also significant (maximum Wilcoxon p=0.0476<0.05).

The parameter settings of DMSCD also affect its processing performance. Specifically, increasing $M_{1}$ and $M_{2}$ can reduce the random fluctuations in noise estimation; a larger $T_{s}$ makes the first-stage denoising result more conservative and the denoising process slower; a larger $R_{s}$ increases the denoising speed, whereas a larger $R_{f}$ accelerates compensation. The ratio of $R_{s}$ to $R_{f}$ jointly determines the attenuation speed; a larger ratio makes attenuation faster. When $M_{1}$ is sufficiently large, an $R_{s}$ closer to 1 provides better denoising, while $R_{f}$ should be smaller than $R_{s}$. A larger $\alpha_{2}$ produces greater amplitude attenuation in the second stage; a larger $g_{\min,2}$ gives a larger minimum amplitude, making it more difficult to completely remove noise; $L_{2}$ (or *h*) is used to smooth the spectrum, and a larger smoothing window makes it easier to merge adjacent spectral peaks. The mean errors obtained by batch processing the constructed waveforms with different algorithm parameters are shown in Figure 11.

Figure 11a shows that increasing $M_{1}$ causes the errors to approach stable values, but also increases PPRE. This is because frequency information is continuously lost during the multiple iterations, weakening the pulse peak. Once $M_{1}$ exceeds 30, all three errors are basically stable.

Figure 11b shows that as $T_{s}$ increases, NRMSE and SRE first decrease and then increase, reaching their optimum values near 1, whereas PPRE decreases monotonically. Considering its practical significance, a value slightly greater than 1 is recommended.

Figure 11c shows that when $R_{s}$ is greater than $R_{f}$, the algorithm can perform stable denoising. In addition, the larger $R_{s}$ is and the larger the ratio of $R_{s}$ to $R_{f}$, the faster the decrease.

Figure 11d shows that all errors gradually decrease as $M_{2}$ increases and become stable once $M_{2}$ exceeds 30.

Figure 11e shows that all three errors increase as $\alpha_{2}$ increases; therefore, a relatively small value should be selected.

Figure 11f–h show that as $g_{\min,2}$ increases, NRMSE and SRE gradually increase, whereas PPRE gradually decreases. However, the waveforms clearly show that a larger $g_{\min,2}$ significantly increases the noise level. Therefore, a relatively small value should be used in practice.

Figure 11i–l show that as $L_{2}$ increases, NRMSE and SRE first decrease and then increase, whereas PPRE gradually increases. This is because actual signals are mostly continuous spectra and do not contain individual frequency bins with high amplitudes. Moderate spectral smoothing can reduce the amplitude of an individual high-amplitude frequency bin while retaining the signal-peak characteristics.

According to the experimental principle, the computational complexity of DMSCD is $O\left(\left(M_{1}+M_{2}+2\right)N\log N+\left(M_{1}+M_{2}\right)N\right)$. The processing times described above were statistically analyzed, and the results are shown in Figure 12. A clear linear relationship can be observed, which is consistent with the theoretical analysis.

## 4. Conclusions

To obtain more accurate time–frequency characteristics of EEMR field strength, this study analyzed the characteristics of EEMR signals and proposed DMSCD for EEMR field-strength measurement, considering their broad spectral distribution, low signal-to-noise ratio, and dispersed noise frequency components. The algorithm uses the noise characteristics of the pre-sampling stage to identify and suppress noise-dominant frequency bins through noise-dominance classification, attenuation, and constrained spectral compensation, and then further suppresses residual noise in overlapping frequency bands through a smoothed spectral gain. The following conclusions were obtained through comparative analysis:

(1) DMSCD can effectively use the signal characteristics of the pre-sampling segment to identify and suppress the influence of broadband noise and retain the spectral characteristics of the signal. It can provide clear spectral-peak distribution information in the frequency domain. Compared with its predecessors, SS and WF, it provides clearly improved NRMSE and SRE performance; compared with baseline algorithms such as VMD, SSA, WD, and AWD, it can identify and suppress noise more effectively.

(2) DMSCD can effectively reduce the influence of noise on the reconstructed field-strength peak. For different antennas at the same time in the same experiment, the average maximum peak-to-peak error of the DMSCD processing results was 74%, lower than the 120% obtained without denoising and the results of all baseline algorithms except SS, and slightly higher than the 72% obtained with SS.

(3) The EEMR field strength generated by the explosion of an RDX charge with a mass of 10 g, a density of 1.6 g/cm^3^, and dimensions of Φ20 × 20 mm has multiple spectral peaks in the range of 0–1 GHz. The main spectral peaks are located near 40, 126, 221, 270, 388, and 675 MHz, among which 160–337 MHz is the main frequency component of EEMR.

## Figures and Tables

**Figure 1:**
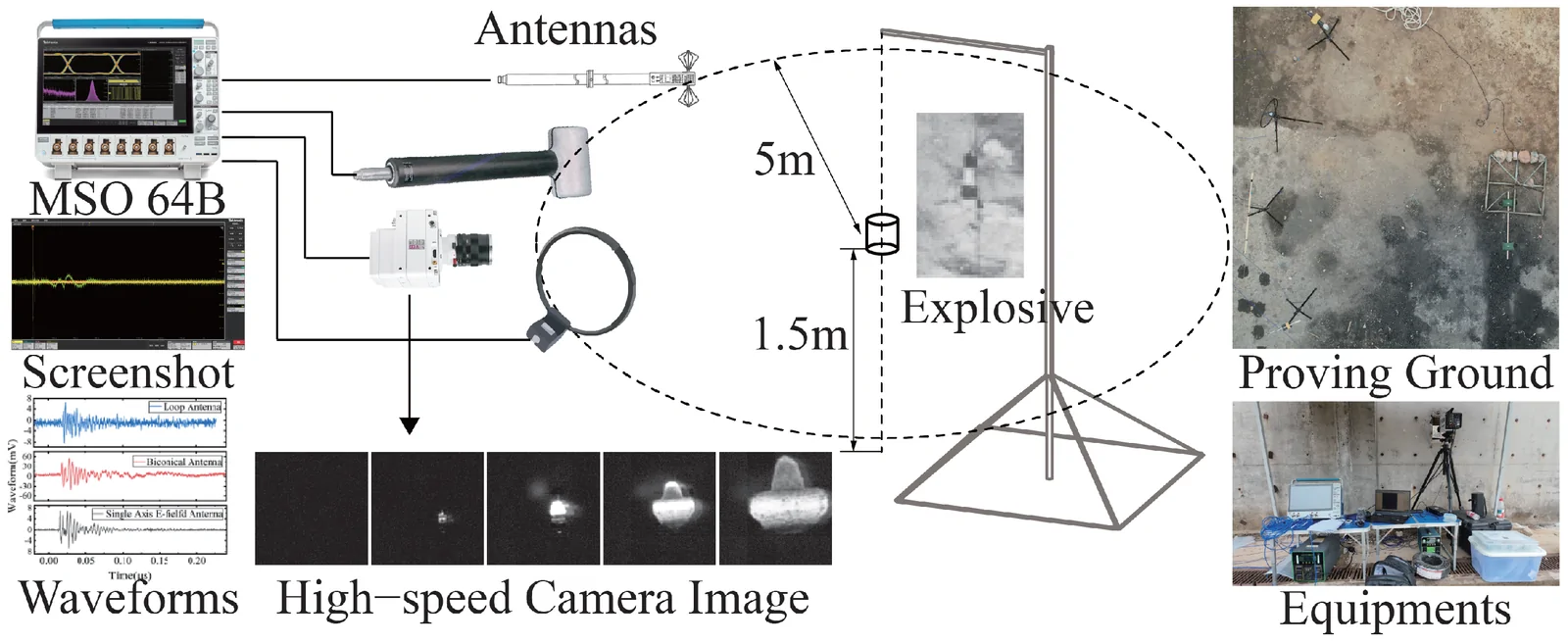
Layout of the EEMR measurement system.

**Figure 2:**
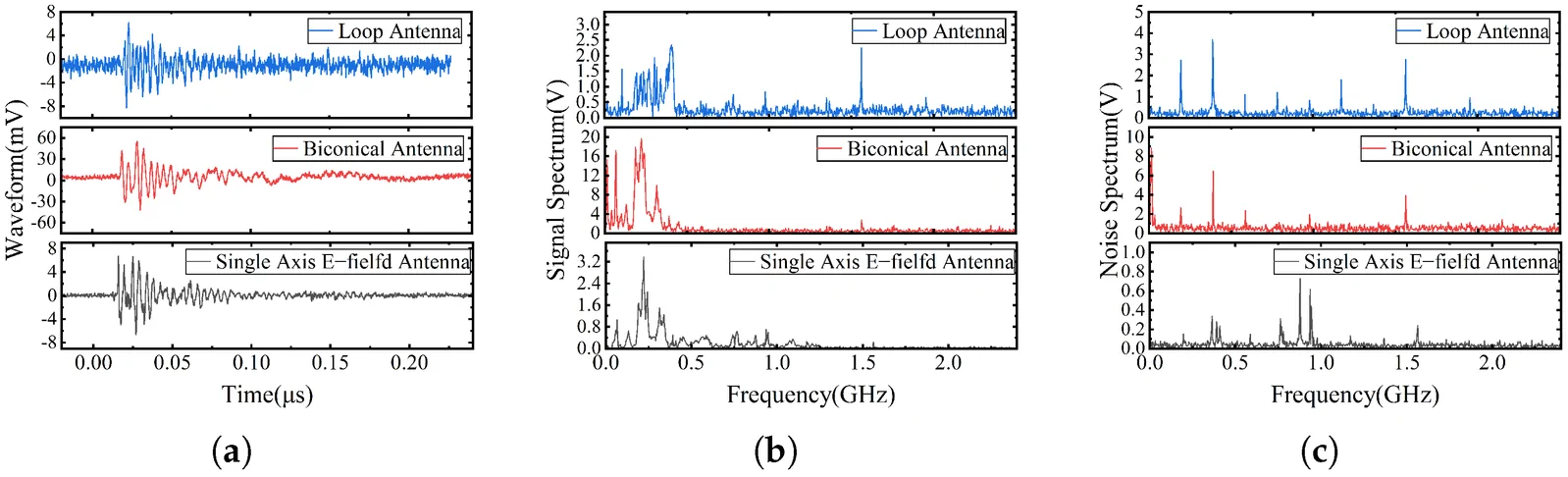
Schematic of the measured EEMR waveform and spectral characteristics. (**a**) Measured waveform. (**b**) Signal-segment spectrum. (**c**) Pre-sampling segment spectrum.

**Figure 3:**
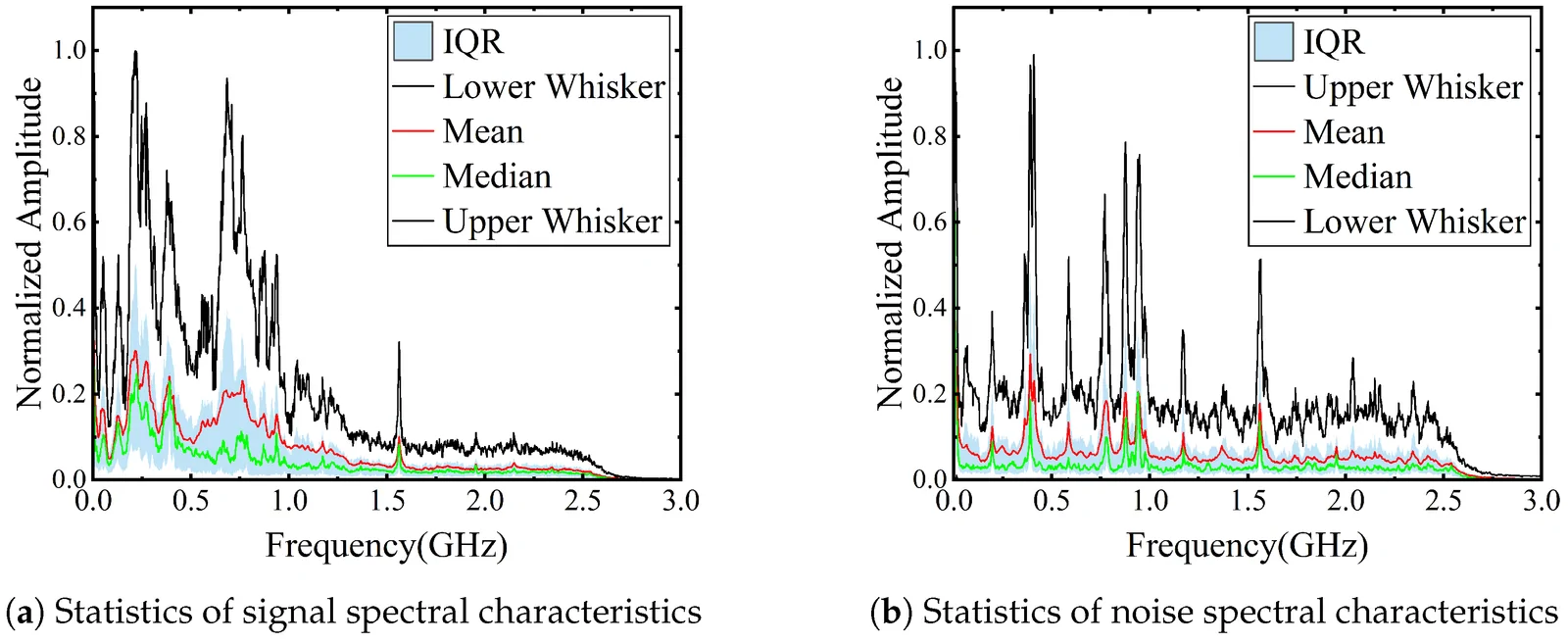
Statistics of the spectral characteristics of the signal and noise.

**Figure 4:**
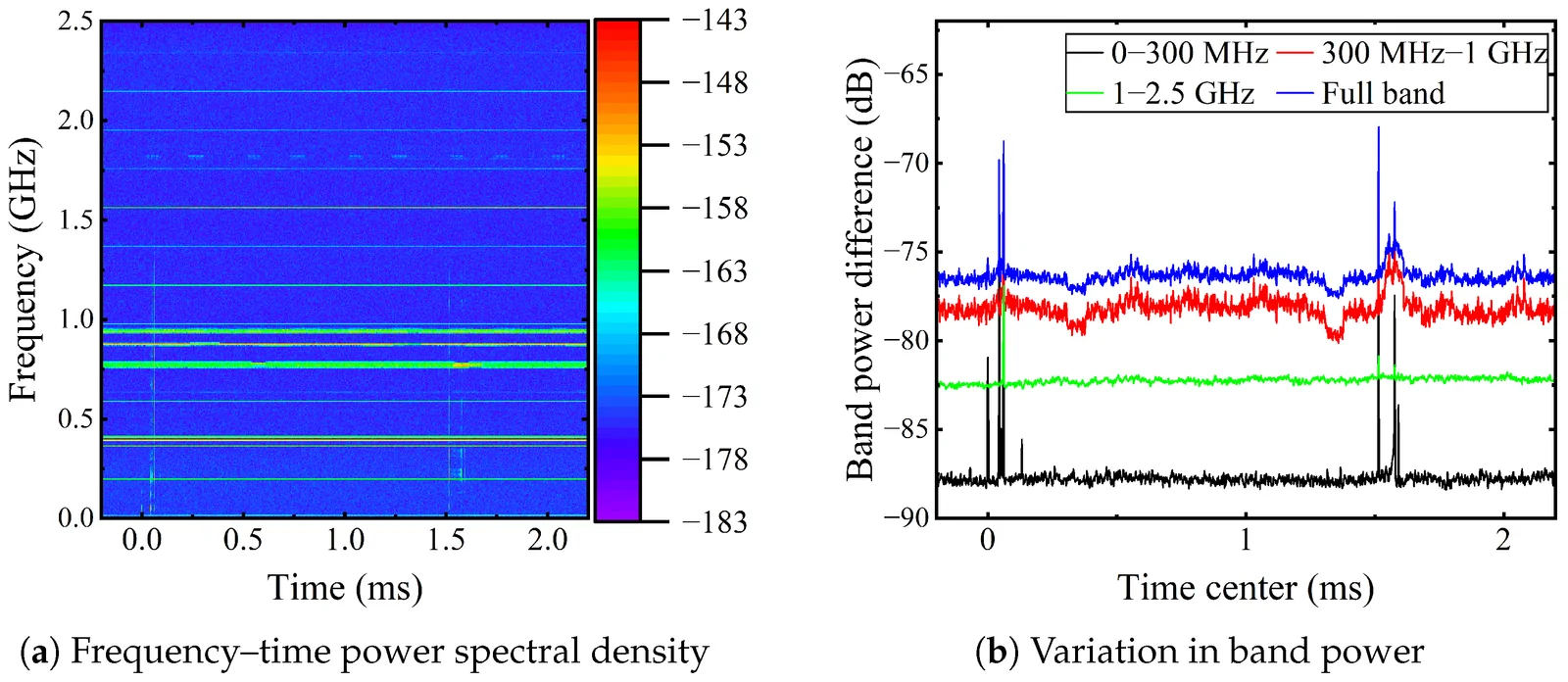
Noise-stability analysis results.

**Figure 5:**
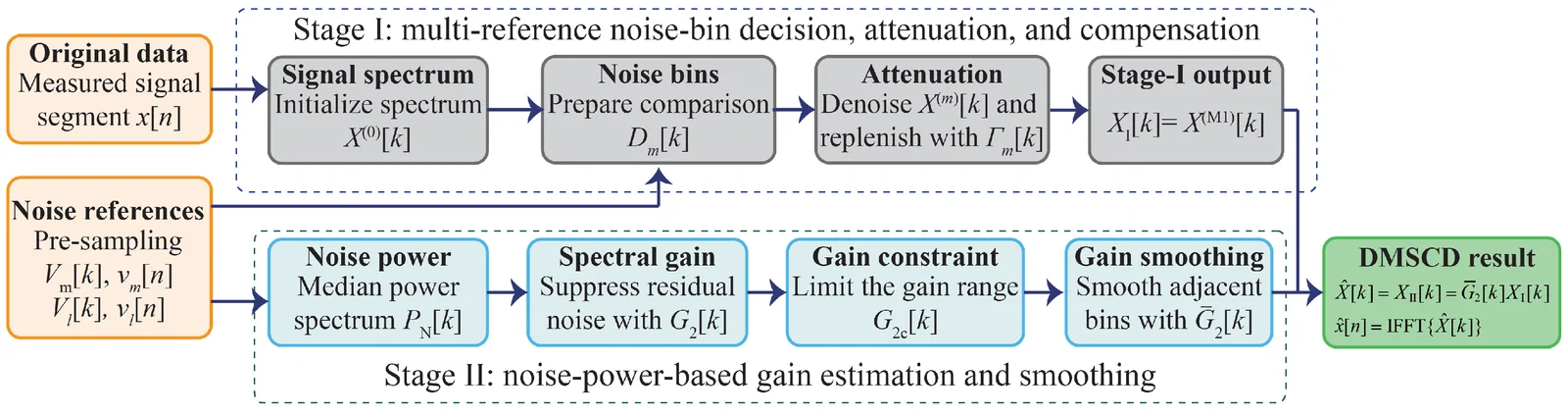
Flowchart of the DMSCD algorithm.

**Figure 6:**
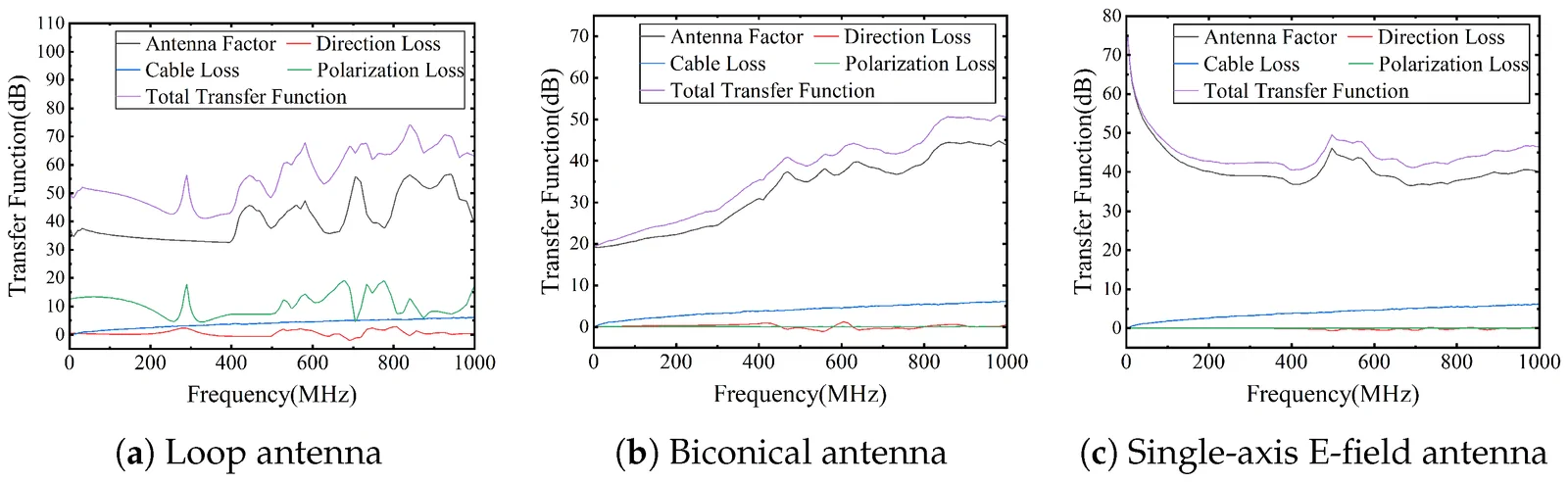
Frequency-response characteristics of the antenna measurement systems.

**Figure 7:**
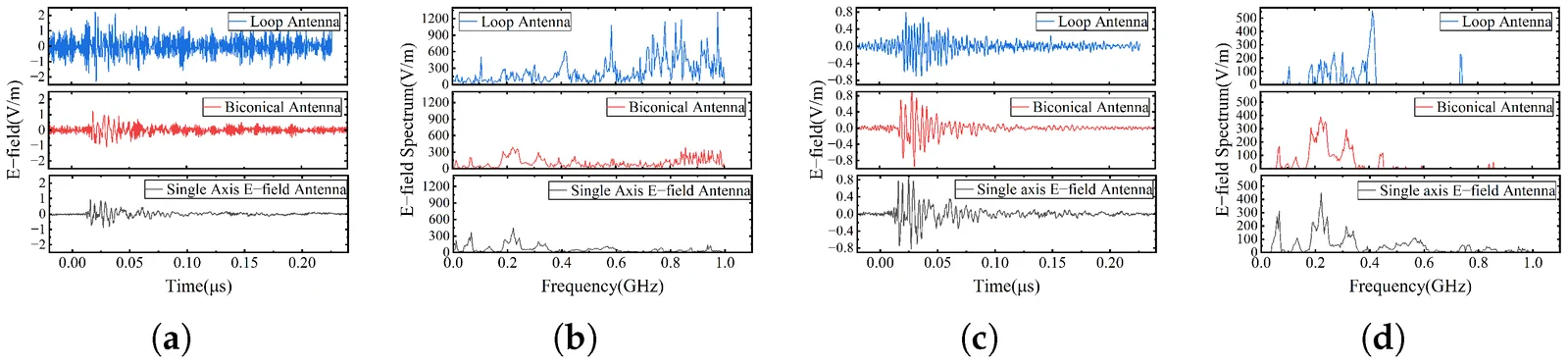
Consistency analysis of the processing results obtained by different antennas in the same experiment. (**a**) Reconstructed E-field waveform without denoising. (**b**) Reconstructed E-field spectrum without denoising. (**c**) Reconstructed E-field waveform using DMSCD. (**d**) Reconstructed E-field spectrum using DMSCD.

**Figure 8:**
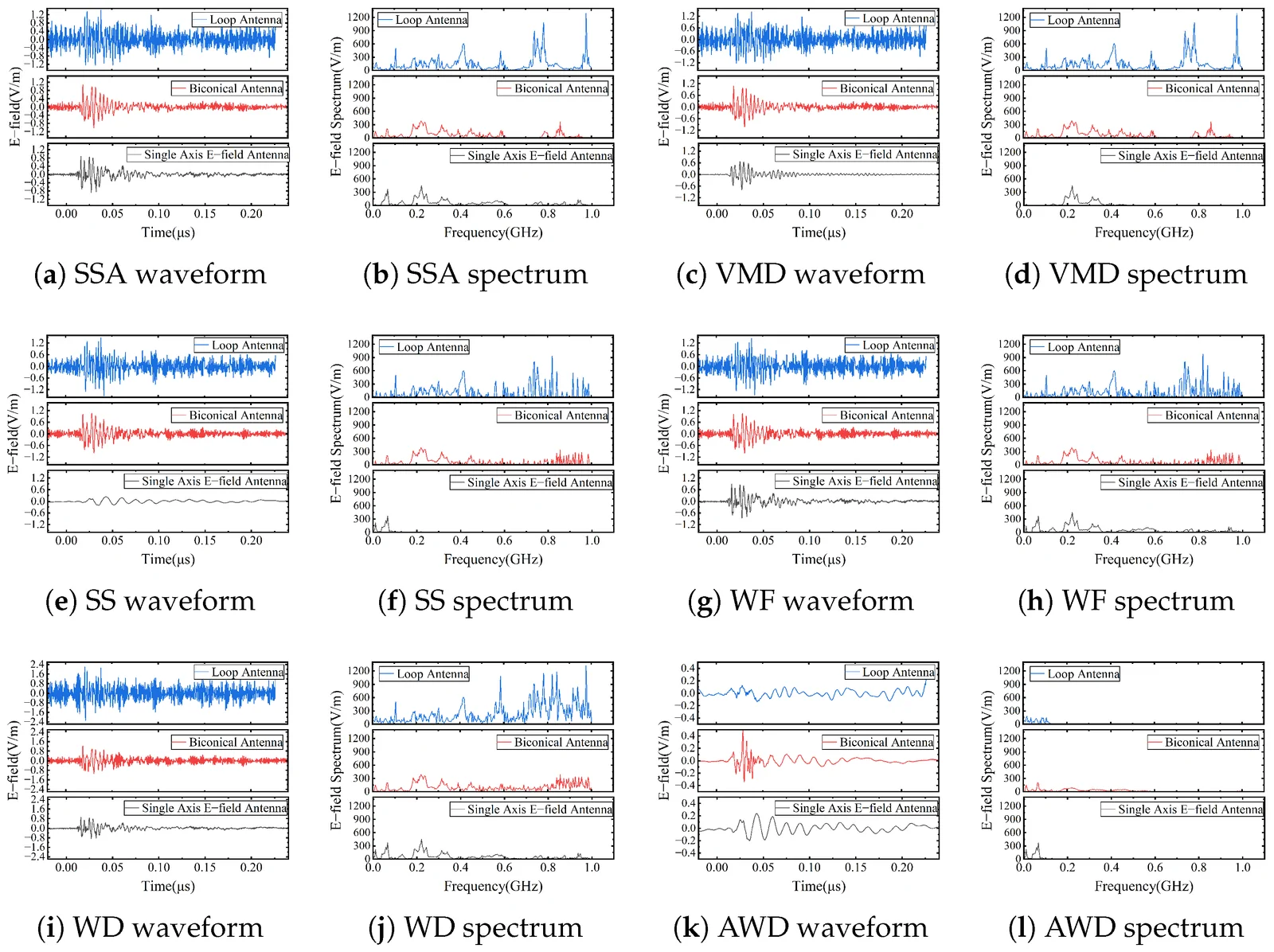
Comparison of the processing results obtained with the baseline algorithms.

**Figure 9:**
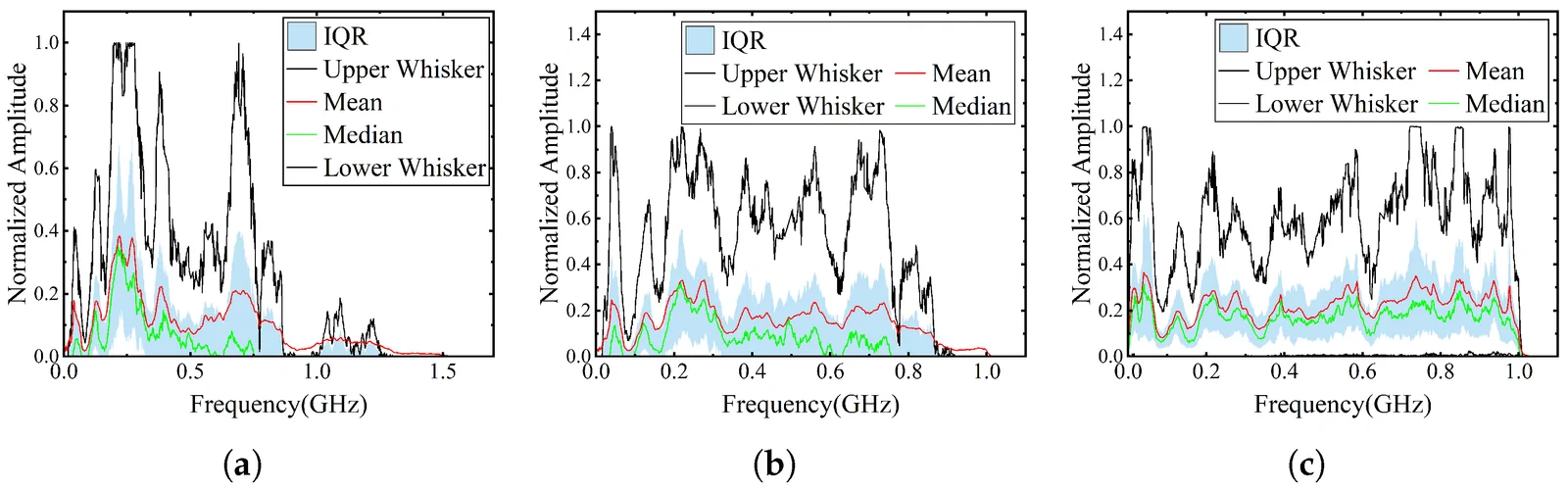
Statistical comparison of signal and reconstructed electric field-strength spectra with and without DMSCD. (**a**) Signal spectrum (DMSCD). (**b**) Field-strength spectrum (DMSCD). (**c**) Field-strength spectrum (raw).

**Figure 10:**
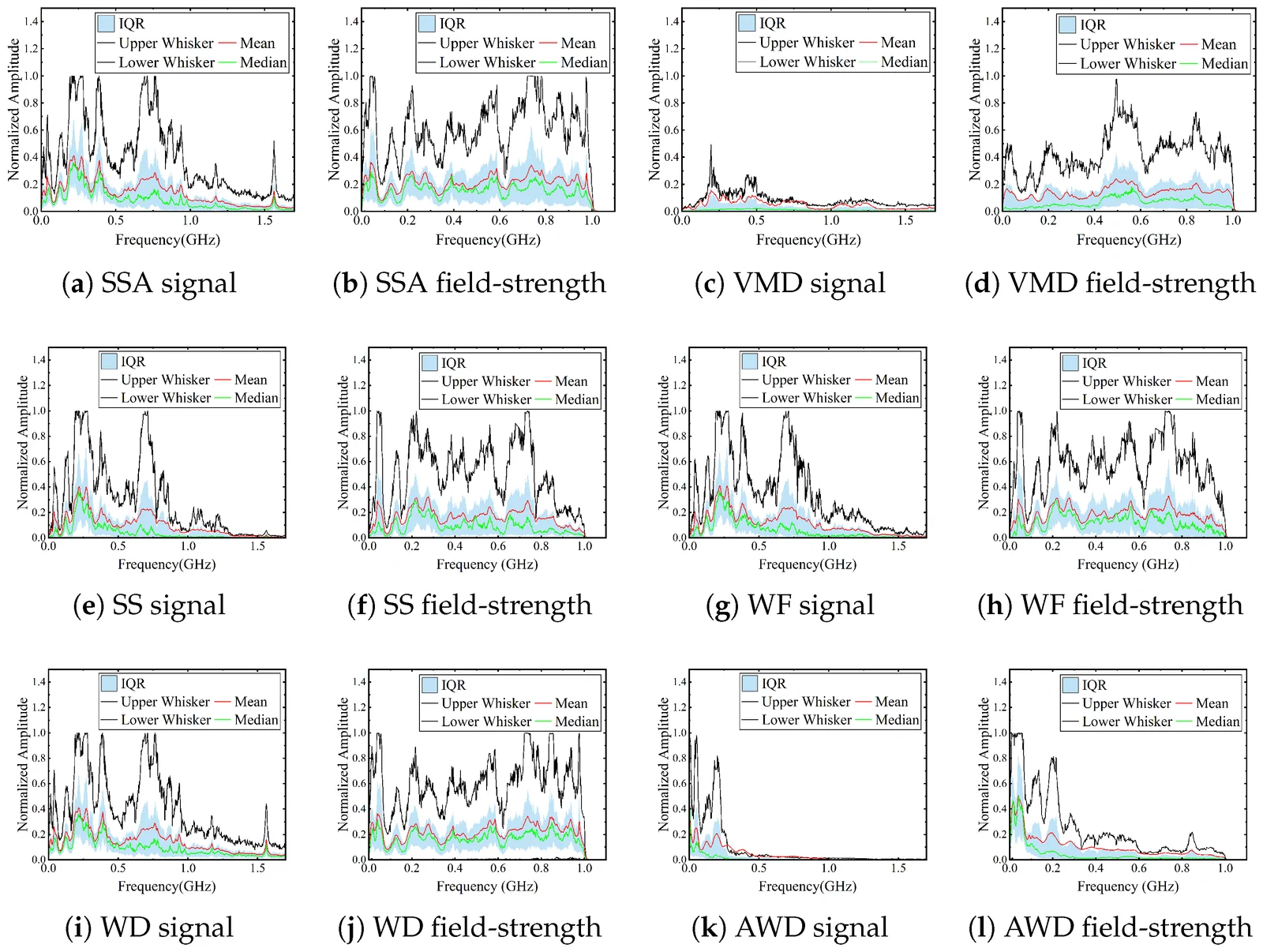
Statistical spectral results obtained with the baseline algorithms.

**Figure 11:**
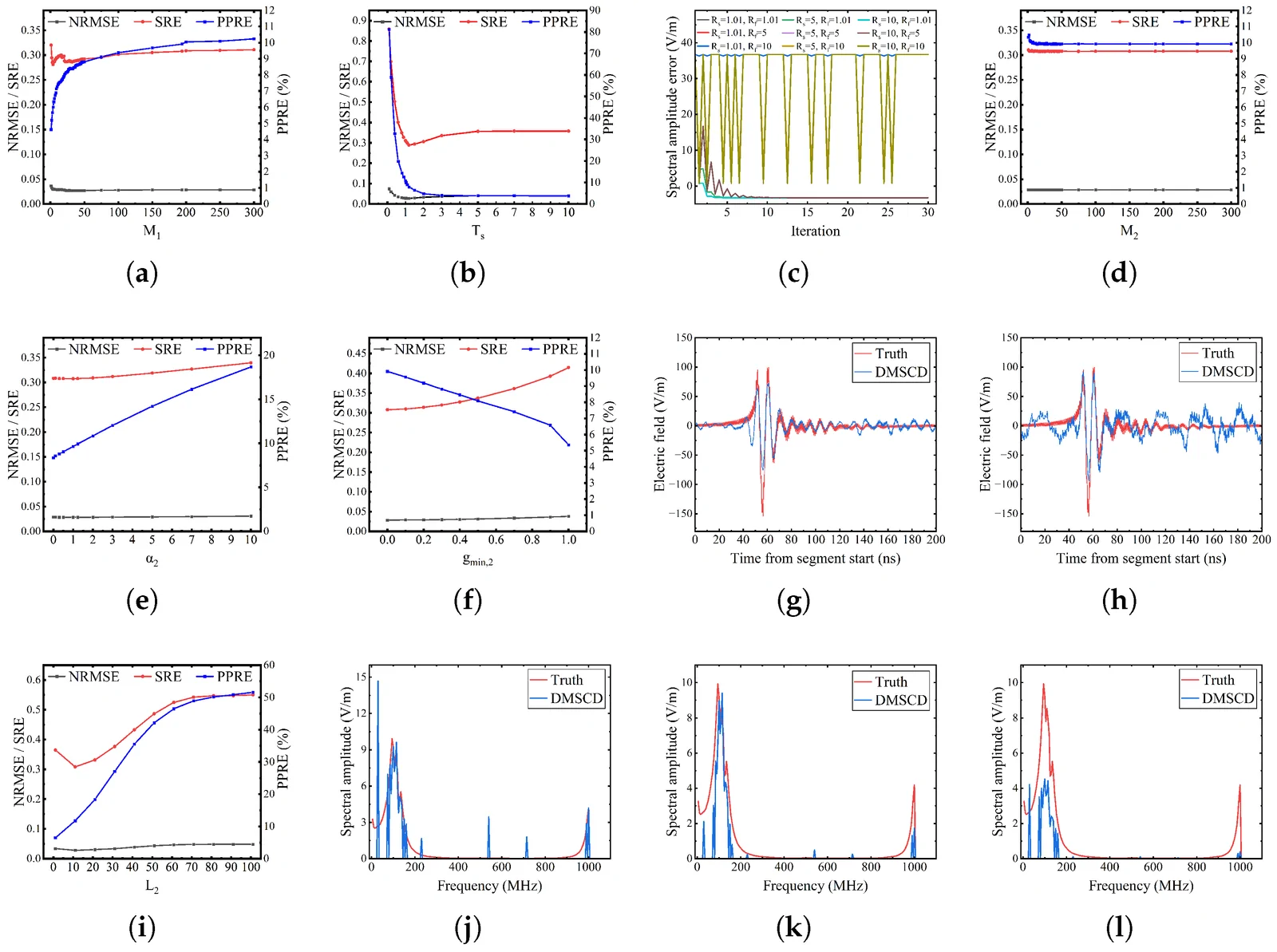
Effect of DMSCD parameters on the denoising results. (**a**) Effect of $M_{1}$ on the three error metrics. (**b**) Effect of $T_{s}$ on the three error metrics. (**c**) Noise spectral-amplitude error for different $R_{s}$ and $R_{f}$. (**d**) Effect of $M_{2}$ on the three error metrics. (**e**) Effect of $\alpha_{2}$ on the three error metrics. (**f**) Effect of $g_{\min,2}$ on the three error metrics. (**g**) Waveform at $g_{\min,2}\approx 0.0005$. (**h**) Waveform at $g_{\min,2}=1$. (**i**) Effect of $L_{2}$ on the three error metrics. (**j**) Spectrum at $L_{2}=1$. (**k**) Spectrum at $L_{2}=7$. (**l**) Spectrum at $L_{2}=31$.

**Figure 12:**
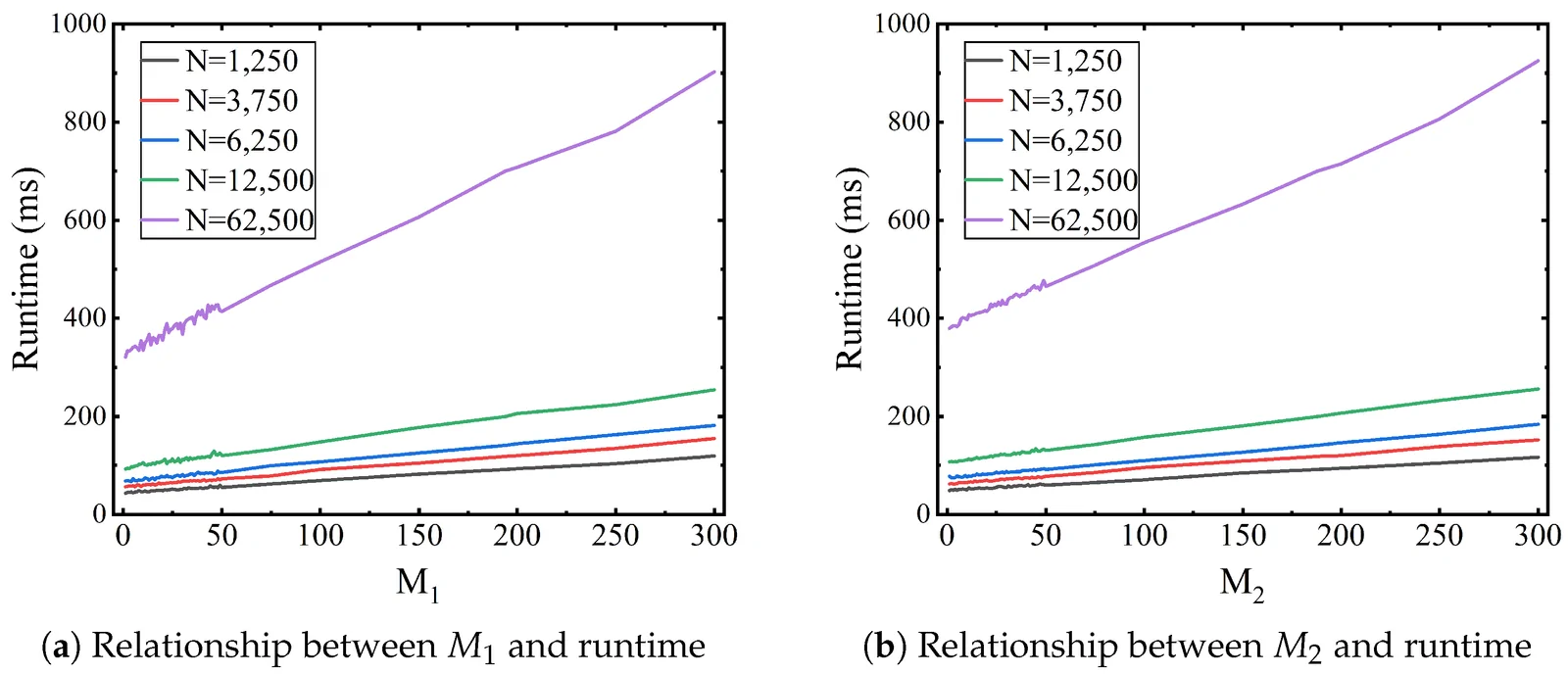
DMSCD runtime statistics.

**Table 1:** Statistics of the peak-to-peak errors of the processing results.

Algorithm	Maximum Error	Minimum Error	Average Error
No denoising	173%	77%	120%
VMD	112%	90%	97%
SSA	172%	12%	91%
DMSCD	164%	26%	74%
SS	137%	15%	72%
WF	151%	36%	77%
WD	184%	77%	116%
AWD	351%	10%	159%

**Table 2:** Comparison of field-strength reconstruction performance of different algorithms under different noise conditions.

Metric	Algorithm	Uniform Noise	Gaussian Noise	Measured Noise	All Noise
NRMSE	DMSCD	0.0530	0.0616	0.0309	0.0441
	VMD	0.1177	0.1611	0.1004	0.1199
	SSA	0.2118	0.2388	0.0639	0.1446
	SS	0.0844	0.1603	0.0424	0.0823
	WD	0.2522	0.3318	0.0730	0.1825
	WF	0.0746	0.1441	0.0383	0.0738
	AWD	0.1754	0.2882	0.1013	0.1665
SRE	DMSCD	0.5531	0.6561	0.2630	0.4338
	VMD	1.2067	1.7394	0.8725	1.1728
	SSA	2.3261	2.6108	0.4874	1.4779
	SS	0.8784	1.7481	0.3292	0.8212
	WD	2.7573	3.6616	0.5509	1.8802
	WF	0.7697	1.5630	0.2947	0.7305
	AWD	1.8787	3.1762	0.7811	1.6543
PPRE	DMSCD	19.26%	31.64%	5.48%	15.46%
	VMD	23.86%	38.93%	75.09%	53.24%
	SSA	39.21%	39.25%	5.59%	22.41%
	SS	11.01%	33.47%	2.69%	12.46%
	WD	37.17%	43.47%	4.80%	22.56%
	WF	10.30%	28.94%	2.60%	11.11%
	AWD	25.48%	41.75%	29.40%	31.51%

## Data Availability

The data supporting the findings of this study are available from the corresponding authors upon reasonable request.

## Abbreviations

The following abbreviations are used in this manuscript:

EEMR	Explosion electromagnetic radiation
DMSCD	Dual-stage multi-reference spectral-subtraction compensation denoising
VMD	Variational mode decomposition
SSA	Singular spectrum analysis
SS	Spectral subtraction
WF	Wiener filtering
WD	Wavelet denoising
AWD	Adaptive wavelet denoising
NRMSE	Normalized root-mean-square error
SRE	Spectral relative error
PPRE	Peak-to-peak relative error
FFT	Fast Fourier transform
IFFT	Inverse fast Fourier transform
